# Cosmetic and functional outcomes of excisional surgical wounds healed by secondary intention: A systematic review

**DOI:** 10.1016/j.jdin.2025.05.016

**Published:** 2025-07-05

**Authors:** Jalal Maghfour, John Meisenheimer, Jonathan Kantor

**Affiliations:** a Department of Dermatology, Henry Ford Health, Detroit, Michigan; b Department of Dermatology, University of Minnesota, Minneapolis, Minnesota; c Department of Engineering Science, University of Oxford, Oxford, UK; d Florida Center for Dermatology, St Augustine, Florida

**Keywords:** cosmetic outcome, cutaneous defect, epidemiology, repair, secondary intention healing, second intention healing, SIH

## Abstract

**Background:**

Data regarding short-term and long-term cosmesis and functional outcomes of excisional surgical wounds healed by secondary intention healing (SIH) are limited.

**Objective:**

To conduct a systematic review and assess the cosmetic and functional acceptability of SIH for acute excisional surgical wounds.

**Methods:**

Full-text articles queried from PubMed and Embase databases between January 1964 and April 2024 with cosmetic outcome data of human subjects with acute surgical wounds healed by SIH were included. Preferred Reporting Items for Systematic reviews and Meta-Analyses guidelines were followed.

**Results:**

A total of 1655 surgical wounds, of which 1518 (91.7%) healed by SIH, from 35 studies, were included in this review. The most frequent indication for SIH was a defect resulting from excision of nonmelanoma skin cancer (keratinocyte carcinoma), which was identified in 1439 (86%) of patients. Common sites for SIH included the nose (23.3%), periocular region (15.46%), and forehead (13.5%). The majority of wounds on the forehead, medial canthus, lower eyelid, nasal ala, cheeks, lips, postauricular area, and feet resulted in good to excellent cosmetic results, whereas those on the scalp, nasal dorsum, nasal tip, nasal sidewall, and chin yielded less acceptable cosmetic results. Given the baseline variability in cosmesis of primarily closed wounds in some anatomic locations, however, these data suggest the need for future prospective studies.

**Summary:**

SIH may produce an acceptable cosmetic and functional outcome for selected defects and may be of clinical benefit in the appropriate setting. This must be weighed against the potentially improved cosmesis and more rapid healing seen with primarily closed defects.

**Capsule Summary**

• Anatomic location is known to influence the healing outcome of wounds healed by secondary intention.
• Wound size and depth may impact the cosmetic appearance of wounds healed by secondary intention located in specific anatomic subunits such as the nasal ala, upper eyelid, temple, and antihelix, while age may not be associated with cosmetic outcomes. Future prospective studies may be warranted.

## Introduction

Surgical excision of benign and malignant lesions results in a variety of cutaneous defects benefitting from various degrees of tissue reconstruction.^1^ Optimal reconstruction of skin defects aims to close the defects with good cosmesis and, most importantly, without functional complications.^1^ Wound management encompasses either surgical repair (including primary linear closure, skin grafts, local, regional, and occasionally free flaps) or secondary intention healing (SIH), defined as wounds allowed to heal without an interventional surgical closure.^1^ SIH occurs when wound edges are not approximated and instead left open to heal through the development of granulation tissue. Healing by secondary intention (SI) has been a valuable method of wound management for centuries, although it may be significantly slower than closing a wound primarily.^1^

When Mohs Micrographic Surgery was first developed, the majority of wounds were left to heal by SIH, and this approach, in the appropriate clinical context, has been shown to yield acceptable cosmetic outcomes.^2^, ^3^, ^4^ One of the most clinically important factors in predicting the outcome of healed wounds is anatomic location.^5^ SIH of wounds located on the concave surfaces of the nose, eye, ear, and temple regions have been reported to result in optimal functional and cosmetic outcomes, while SIH of wounds on convex surfaces may lead to undesirable functional and cosmetic outcomes.^5^ Other areas have been reported to heal with variable results, including the forehead, antihelix of the ear, lips and cheeks, and eyelid.^4^

With the introduction of fresh tissue technique in Mohs Micrographic Surgery, immediate surgical closure of complex wounds became the preferred method of wound management. However, SIH may still be considered for selected motivated patients willing to undergo the longer healing times, although data on the cosmetic outcomes associated with this approach are lacking.^6^ While a range of suturing techniques and repair options have been explored for surgical wounds—largely focusing on reduction of tension across the wound surface during the healing process^7^—little is known regarding optimal approaches to secondary wound healing and, most importantly, wound choice for this approach. We therefore aimed to systematically review the evidence for the use of SIH in the management of acute surgical wounds, with a primary goal to examine the cosmetic outcomes of wounds healed by SI.

## Methods

A comprehensive literature review was performed according to the Preferred Reporting Items for Systematic reviews and Meta-Analyses reporting guidelines.^8^ PubMed and Embase databases were queried for the period of January 1964 to April 2024, using combinations of the following key terms: “secondary intention healing”, “SIH”, “acute surgical wounds”, “secondary intention”, and “nonmelanoma skin cancer.” The search strategy and approach were developed with the help of a medical research librarian. Two independent reviewers (J.M. and J.K.) independently assessed the eligibility of identified reports. The inclusion criteria were observational or interventional studies that reported cosmetic outcome of human subjects with acute surgical wounds, defined as wounds less than 6 weeks old, which were left to heal via SIH. With the exception of review articles, all published reports (eg, case studies, case reports, and series) with a minimum number of 1 patient were included. Studies were excluded during the title and abstract screening for the following reasons: (1) nonhuman studies; (2) articles written in languages other than English, French, and Spanish; (3) articles reporting outcomes of wounds that arose from nonsurgical causes (eg, traumatic lacerations, infections, carbon dioxide laser ablative surgeries, electrosurgery) or those that were chronic (>6 weeks) such as excision of hidradenitis suppurativa lesions; and (4) articles with no cosmetic outcome data. Given that majority of studies used a nominal scale to assess cosmetic outcome, data synthesis was performed by grouping together similar scales (eg, excellent, good, acceptable).

The quality assessment of identified reports was performed according to the guidelines provided by the Centre for Evidence-Based Medicine at Oxford. The included studies were classified based on the “Therapy/Prevention, Aetiology/Harm” categories with 1a being the highest quality and 5 being the lowest quality.^8^

### Study selection

**Fig 1:**
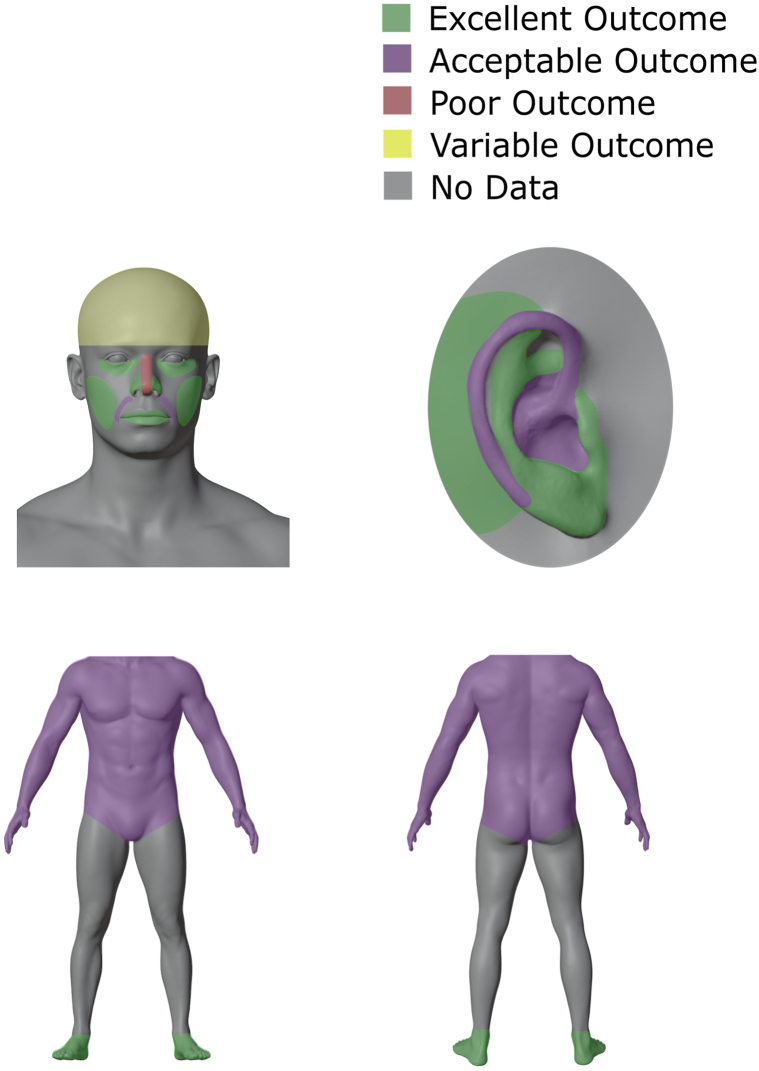
Outcome of wounds healed by secondary intention.

A total of 333 studies were identified, of which 279 were nonduplicated reports. All articles were initially screened by reviewing the abstract. Of the 279 reports, 68 (25.7%) underwent full-text review (Fig 1).

### Quality assessment

According to the guidelines provided by the Centre for Evidence-Based Medicine at Oxford,^8^ 2 studies were graded as 2A, 2 studies were graded as 2b, 6 studies were graded as 3A, 17 studies were graded as 3b, 8 studies were graded as 4b, and 2 studies were graded as 5. All classifications represent evidence of moderate to low quality based on study designs.

## Results

Thirty-five published reports were included,^1^^,^^4^^,^^9^, ^10^, ^11^, ^12^, ^13^, ^14^, ^15^, ^16^, ^17^, ^18^, ^19^, ^20^, ^21^, ^22^, ^23^, ^24^, ^25^, ^26^, ^27^, ^28^, ^29^, ^30^, ^31^, ^32^, ^33^, ^34^, ^35^, ^36^, ^37^, ^38^, ^39^, ^40^, ^41^ of which 28 were cohort studies and 2 were randomized controlled trials. A total of 1655 surgical wounds from 1673 patients (age range: 18-95 years) were identified, of which 1518 (91.7%) were allowed to heal by SIH. The most frequent indication for SIH was defects resulting from excision of keratinocyte carcinoma, which was identified in 1439 (86%) patients. The most common anatomic sites included the nose (354 [23.3%]), periocular area (233 [15.46%]), and forehead (224 [13.5%]). These were closely followed by the ear (204 [12.3%]), scalp (113 [6.83%]), and lip (71 [4.53%]).

### Face

A total of 1023 wounds were located on the face. The most common anatomic sites healed by SI included the nose (354 [34.6%]), periocular area (233 [22.7%]), and forehead (224 [21.9%]). Cosmetic outcome varied according to anatomic area (Table I). We also identified 135 wound defects on the face (unspecified subunit) of which 37 (27.4%) healed by SI.^4^

**Table I:** Cosmetic outcome of wounds on the face that healed by secondary intent

Lesion size (cm^2^)	Wound healing time (weeks)	Cosmetic outcome	Additional findings
Forehead, (244, 23.8%)			
0.09-1.09	2-6	Median: good (42/70) Glabella: good-excellent (10/1) Paramedian/lateral: excellent (5/5) Temple wound: good-excellent (57/60) Brow and suprabrow: good-excellent (16/19)	Depressed scars for deeper wounds
Periocular area, unspecified (17, 1.66%)			
0.25-2.1	PSG: 11.0 ± vs 6.9 days vs SIH: 30.4 ± 13.3 d	Periocular: excellent; no significant difference between SIH and primary closure	Mild hypertrophy, overgranulation, notching of eyelid, epiphora, and ectropion were commonly reported
Upper eyelids (17, 1.66%)			
1.4-17.19	1.4-17.19	Upper eyelids: SIH: poor: (9 [82%]) and periosteum (3 [100%]) Primary surgical closure: poor: 5/9	Atrophic scarring and dimpling were commonly reported deformities
Lower eyelids (71, 6.9%)			
0.9-1.09	2-6	Lower eyelids: good to excellent (65 [92%])	Five patients had mild notching
Canthus (117, 11.4%)			
0.25-2.1	2-6	Medial canthus: excellent 54% Lateral canthus: excellent 60%	Reported complications of medial canthus wounds include cicatricial ectropion, and canthal webbing which were observed in 6 patients
Nasal ala (112, 10.9%)			
0.25-2.1	2-6	Nasal ala: excellent (80 [71.6%]) MMSS scar quality: optimal in 17 (53.2%) of wounds	Scar contracture resulting in obstructive breathing in 1 patient
Nasal tip (99, 9.6%)			
1.06-4	2-12	Nasal tip: Poor: 95/99 (95.7%)	Pinched and depressed scar was commonly reported as an undesirable cosmetic outcome
Nasal sidewall (52, 4.84%)			
<1 to >2 cm	3-8	Nasal sidewall: Good: 18/40 (45%) with scar scale score of 9.5 (considered optimal) Poor: 22/40 (55%)	Depressed scar
Dorsum of the nose (37, 3.4%)			
0.7-4	2-12	Dorsum of the nose: a mean MSS score greater than 10.8 (defined as suboptimal). Average: 3/12 (25%) Excellent: 1/12 (8.3%)	None
Cheeks (16, 1.5%)			
1.5-3.5	2.5-4.5	Good to excellent: 16/16 (100%)	No functional complications were reported
Nasolabial folds (33, 3.2%)			
0.63-11.18	Not reported	Acceptable: 26/28 (93%) Acceptable results were higher in the SIH group compared to the primary closure group (27/28 [96%] vs 9/17 [52.9%])	Scarring and distortion
Lip, upper (50, 4.8%)			
0.30-1.35	3-6	Alar base: excellent: 7/7 (100%) Lip proper: acceptable: 15/15 (100%) Upper lip wounds within 2 mm of the vermillion: unacceptable: 11/20 (50%) Upper lip within 3 mm of the vermillion: acceptable: 3/3 (100%)	Superficial scarring was reported for wounds involving lip proper Distortion of the vermilion border was noted for wounds within 2 mm of vermillion border
Apical triangle, (24, 2.3%)			
0.3-1.35	1-2	Apical triangle: no significant differences were noted in cosmetic outcome between surgical closure and SIH (VAS, 8.23 ± 0.96 vs 7.78 ± 1.52, surgery vs SIH, respectively; *P* value = .5267)	Not reported
Lower lip (21, 2.0%)			
0.90-1.20	3-5	Lower lip: good to excellent: 11/21 (52.3%) Cutaneous lip and vermilion: poor: 7/21 (33.3%) Wounds confined to the vermilion: acceptable to excellent: 3/21 (14.2%)	Not reported
Philtrum (8, 0.78%)			
0.9-1.09	2-6	Philtrum: good to excellent: 7/8 (87.5%)	Five patients had mild notching of the lower eyelid.

*MMSS*, Mean Manchester Score Scale; *MSS*, Manchester Score Scale; *PSG*, primary surgical group; *SIH*, secondary intention healing; *VAS*, visual analog scale.

### Forehead

SIH of 224 wound defects were identified, of which 180 (80.3%) had follow-up data.^10^^,^^12^^,^^14^^,^^18^^,^^35^^,^^36^ Central/median forehead (70 [38.8%]) was the most common anatomic subunit left to heal by SI.

This was followed by wounds on the temple (57 [95%]), glabellar, and paramedian and lateral subunits (5 [100%]). Of these anatomic subunits, wounds located on the temple, brow, and suprabrow healed with good to excellent cosmetic results (92%); these included defects that were large and deep. Wounds involving the muscle, periosteum, and bone (*n* = 4) resulted in a poor cosmetic outcome.

Of 70 wounds on the central/median forehead, 42 (60%) produced a good cosmetic outcome, whereas 28 resulted in a poor cosmetic outcome with scars being described as atrophic and depressed. SIH of glabellar wounds (12) produced optimal cosmetic outcome with a good to excellent result observed in 88.9% of lesions. Wounds involving the paramedian and lateral subunits also produced excellent cosmetic results (5).

A ‘good’ to ‘excellent’ result was reported in 57 (95%) of temple wounds. There were 40 wounds in the temporal subunit that were large and deep, of which 38 healed with good cosmetic results. The majority of brow and suprabrow wound defects (84%) healed with good to excellent cosmetic results. Some of these defects were considered large and deep. For most patients, minimal brow distortion was observed.

### Periocular area

A total of 233 wound defects involving the periocular area were included. Medial canthus (104 [39.1%]) was the most common subunit allowed to heal via SI.^1^^,^^11^^,^^12^^,^^18^^,^^28^^,^^36^ This was closely followed by lower eyelids (71 [26.7%]),^1^^,^^14^^,^^28^ upper eyelids (17 [6.4%]),^10^^,^^18^^,^^42^ lateral canthus and palpebral angle (13 [4.88%]), and eyebrows (11 [4.13%]). There were 50 wounds with unspecified anatomic subunits, of which 17 (7.3%) healed by SI.^34^

Wounds on the medial (54%), lateral (60%) canthus, and lower eyelid (92%) produced excellent results. An acceptable cosmetic outcome was noted in 82% of upper eyelids wounds.

Commonly reported complications included cicatricial ectropion and canthal webbing, which were observed in 6 patients.

### Nose

Within the topographic anatomy of the nose, the nasal ala (112 [31.6%]) was the most common subunit left to heal by SI.^20^^,^^35^^,^^36^ This was followed by nasal tip (99 [27.9%]),^11^^,^^12^^,^^18^^,^^23^^,^^27^^,^^42^ nasal sidewall (52 [14.68%]),^12^^,^^23^^,^^27^^,^^42^^,^^43^ and the dorsum of the nose (37 [10.45%]).^18^^,^^20^^,^^23^^,^^27^^,^^42^

There were 54 wound defects involving various anatomic areas of the nose (wing 11 [26.2%], columella 5 [11.9%], central nose 5 [11.5%], lateral nose 7 [16.6%], nose, unspecified anatomic subunit 12 [4.76%], alar crease 7 [12.9%], nostril [5.55%], root 1 [1.85%], and nasojugal fold 3 [5.55%]).^1^^,^^11^^,^^12^^,^^17^^,^^18^^,^^41^

Within the anatomic subunit of the nose, most wounds located on the nasal ala (71.6%) produced an excellent cosmetic outcome, followed by wounds located on the nasal sidewall (45%). Wounds located on the nasal tip (95.7%) and dorsum of the nose (66.6%) resulted in suboptimal healing. Acceptable cosmetic outcome was noted in 93% of wounds on the nasolabial folds.

Although majority of wounds on the nasal tip resulted in suboptimal outcomes, authors from a retrospective study found a strong correlation between defect depth and scar outcome (assessed using the Manchester scar scale), with superficial wounds yielding optimal results (Manchester scar scale of 6.5 cm for superficial dermis).^23^

Comparative cosmetic outcome data between SIH and primary closure were available for 45 nasolabial fold wounds.^9^ Compared to primary closure, a higher proportion of wounds healed by SI was rated as acceptable (SIH group: 27/28 [96%] vs primary closure: 9/17 [52.9%]). Subjective assessment of these wounds revealed a superior cosmetic result with SIH versus primary closure.

Commonly reported sequelae of SIH were scarring and distortion of the nasolabial folds, and obstructed breathing attributed to scar contracture of the nasal ala. For wounds on the nasal tips, pinched and depressed scar were commonly reported as undesirable cosmetic outcomes.

### Lip

Cosmetic outcome of lip defects (*n* = 71) varied according to anatomic subunit (Table I).^9^^,^^13^^,^^42^ SIH was assessed in 50 and 21 wounds involving the upper and lower lip, respectively (Table II). In addition, 24 wound defects involving the apical triangle were included of which 9 (37.5%) healed by SI after partial closure (Table I).^30^

**Table II:** Cosmetic outcome of wound defects located on the head and neck that healed by secondary intention

Lesion size (cm^2^)	Time to wound healing (weeks)	Cosmetic outcome	Additional findings
Scalp (109, 33%)			
1.10-72.25	7-13	Fair (mean VAS of 83) to good (mean VAS of 27) (26/28 [92%]) Poor: 70 (62.5%)	White and depressed scar
Helix (81, 22%)			
1-10	3-10	Helix: acceptable: 19/34 (56%) The mean VAS for SIH was 82.1 (SD: 7.6), a comparable finding to primary closed wounds (VAS: 78.4, SD = 15.1, *P* value = .28)	Permanent notching “cookie bit” deformity was observed for full-thickness defects involving both soft tissue and cartilage. Avascular necrosis with notching was also observed for wounds with denuded perichondrium (anteriorly and posteriorly) irrespective whether cartilage was intact.
Posterior aspect of the ear (38, 8.3%)			
1.53-15.75	3-10	Auricular: acceptable to excellent: 32/38 (84.2%) Retroarticular: acceptable to excellent: 3/38 (7.9%) Posterior ear: acceptable to excellent: 3/38 (7.9%) Postauricular wounds with exposed cartilage: acceptable to excellent: 16/16 (100%)	Minimal to moderate webbing of the sulcus was observed for 9 postauricular defects involving the postauricular sulcus
Antihelix (26, 7.6%)			
1-7.54	3-10	Antihelix: acceptable to excellent: 24/26 (92.3%)	Due to partial excision of the cartilage, flattening of the antihelix was noted in 4 defects
Tragus (17, 5%)			
2.10-29.25	Within 10 wk	Tragus: excellent: 9/17 (52.9%)	Majority of wounds resulted in an acceptable cosmetic outcome. Wound depth did not correlate with cosmetic outcome
Conchae (15, 4.4%)			
0.63-8.91; 3-10 wk	3-10	Conchae: acceptable: 13/15 (87%) Poor: 2/15 (13.3%)	Web formation in poorly healed wounds
Pinna (4, 1.2%)			
0.49-3.68	3-8	Pinna: excellent to good: 3/4 (75%)	Not reported
Combined subunits (12, 5.8%)			
2.10-35	2-12	Combined subunits: Helix and antihelix: acceptable: 10/12 (83.3%)	Contour deformity noted with exposed cartilage

*SD*, Standard deviation; *SIH*, secondary intention healing; *VAS*, visual analog scale.

Within the topographic area of the upper lip, wound defects on the alar base (7 [100%]) resulted in excellent cosmetic outcome, whereas those involving the mucosal lip (15 [100%]) yielded an acceptable cosmetic outcome. For both subunits, lesions were deep, extending to subcutaneous tissue and orbicularis oris muscle.

Upper lip wounds that were within 2 mm of the vermillion (12 [52.1%]) yielded unacceptable cosmetic outcomes. Since lips do not have bony structure, secondary healing of cutaneous lip defects close to the vermillion border may result in distortion during normal wound contracture.

### Head and neck

#### Scalp

There were 109 (35%) wounds on the scalp (42 [37.2%] vertex, 16 [14.2%] frontal, 7 [6.2%] temporal, 5 [4.42%] parietal, and 39 [34.5%] unspecified anatomic subunit).^10^^,^^12^^,^^31^^,^^35^ In addition, SIH was reported for a wound in an unspecific head and neck location and for 3 wounds involving more than 1 subunit (scalp, temple, and forehead).^12^^,^^35^

Of the 109 wounds, 28 (25.6%) were assessed using visual analog scale (VAS) scoring system, of which 26 (of 28 [92%]) healed with a ‘fair’ (mean VAS of 83) to ‘good’ (mean VAS of 27) cosmetic outcome. For the remaining wounds in which cosmetic outcome data were available, majority (53/78 [62.5%]) of the wounds produced a poor cosmetic outcome.

#### Ear

SIH of 204 ear wound defects (63.4%) were identified, of which 81 (of 204 [39.7%]) were located on the helix/triangular fossa, 39 (19.1%) on the posterior auricular, 26 (12.74%) on the antihelix, 17 (8.33%) on the tragus, 15 (7.35%) on the conchae, 4 (1.96%) on the pinna, and 1 (0.49%) defect with no specific anatomic subunit.^11^^,^^19^^,^^26^^,^^35^^,^^36^^,^^43^ 21 (10.3%) wounds involved more than one anatomic subunit. For wounds on the helix, unacceptable results were observed for full-thickness defects involving both soft tissue and cartilage resulting in a permanent notch “cookie bite” deformity. Avascular necrosis with notching was also observed for wounds with denuded perichondrium (anteriorly and posteriorly), irrespective of whether cartilage was intact. Prophylactic antibiotics were used in 17 cases, of which 12 had exposed cartilage. Wound infection occurred in 3 defects, of which 2 (66.7%) had exposed cartilage (Table II).

Acceptable to excellent cosmetic outcome was noted for wounds on the pinna (88%), the posterior aspect of the auricle (84.2%), and the antihelix (92.3%), whereas an acceptable cosmetic outcome was observed for wounds on the conchae (86%), tragus (87.5%), and combined subunits (helix and antihelix, 83.3%).

Although minimal to moderate webbing of the sulcus was observed for 9 postauricular defects involving the postauricular sulcus, due to the preserved configuration of the auricle, the cosmetic outcome was excellent.

### Other anatomic areas

A total of 178 wounds involving various anatomic areas were left to heal by SI. Of these, lower extremities were the most common anatomic sites (29 [15.8%]). This was closely followed by upper extremities (28 [16.2%]), hands (28 [16.2%]), back (28 [16.2%]), feet (26 [15%]), and nails (26 [15%]). Two wounds were located on the trunk and 11 wounds involved both trunk and limbs (unspecified location)^5^^,^^10^^,^^15^^,^^24^^,^^25^^,^^37^^,^^40^ (Table III).

**Table III:** Cosmetic outcome of trunk, upper, lower, and distal extremities of wound defects that healed by secondary intention

Size (cm^2^)	Time to wound healing (weeks)	Cosmetic outcome	Additional findings
Upper extremities (28, 16%)			
0.4-0.8	3-6	Compared to primary closure, SIH produced relatively comparable cosmetic results (4 mm defect, mean VAS [SD]: SIH: 73.1 [14.3] vs primary closure: 69.7 [19.9]; 8 mm defect, mean VAS [SD]: SIH: 46.7 [24.1] vs primary closure site: 45.5 [19.2])	Pain was reported more commonly for 8-mm biopsies than for 4-mm biopsies (SIH sites, 8 mm vs 4 mm: 58% vs 33%, *P* = .03; primary closure sites, 8 mm vs 4 mm: 45% vs 24%, *P* = .05)
Hands (28, 16%)			
1.5-4.6	3-10	Hands: acceptable-good: 28/28 (100%)	Overgranulation was observed in 12 (50%) wounds. Pain was reported in 6 patients following 1 wk of healing.
Trunk (2, 1.2%)			
0.32-28.6	2-4	The mean VBSAS score for wound on the chest was 5 mean VAS for wound on the nipple was 9	None
Back (28, 16%)			
1-7.54	3-10	SIH produced comparable cosmetic outcome (4-mm defect, mean VAS [SD]: primary suture: 71.6 [17.7] vs SIH: 62.6 [11.3]; 8-mm defect: primary suture 51.4 [16.2] vs SIH: 49.2 [16.7])	None
Lower extremities (29, 17%)			
0.40-12	3-10	Compared to primary closure, SIH produced equivalent cosmetic results (4-mm defect, mean VAS [SD]: SIH: 67.2 [14.8] vs primary closure: 68.4 [17.2]; 8-mm defect, mean VAS [SD]: SIH: 45.4 [17.7] vs primary closure site: 48.1 [16.6]). SIH of wound on calf (*n* = 1) resulted in a low scar quality with a VBSAS score of 9	None
Feet (26, 15%)			
32.6	3-8	A superior cosmetic outcome achieved with SIH compared to skin graft investigators, VBSAS: SIH group 7.85 ± 1.52; vs skin graft group: 10.33 ± 1.10 (*P* < .001), and patients (SIH group: 6.62 ± 1.61 vs FTSG: 3.42 ± 1.24, *P* < .001). Functional outcome was also superior in SIH group compared to skin graft group (VAS: 7.69 ± 0.86 vs 4.58 ± 1.31; *P* < .001) Cosmetic outcome of wounds left to heal by SI was compared to negative wound pressure therapy (NWPT) group. Compared to SIH group, a superior cosmetic outcome was achieved with NWPT (VBSAS score: SIH [2.89 ± 1.36] vs NPWT 5.85 [±2.99], *P* = .02).	Rates of infection were significantly higher in SIH group than in NPWT group.
Nail (26, 15%)			
2.10-35.2	2-10	The mean VBSAS (*n* = 14 wound defects) was 4.6 ± 1.3 Nails: fair to satisfactory: 12/26 (46.2%)	No functional outcome impairment

*FTSG*, Full thickness skin graft; *NPWT*, negative pressure wound therapy; *SD*, standard deviation; *SI*, secondary intention; *SIH*, secondary intention healing; *VAS*, visual analog scale; *VBSAS*, Vancouver Burn Scar Assessment Scale.

A good to excellent cosmetic outcome was observed for more than half (66%) of wounds on the lower extremities, nails, and feet. Higher rates of infection were noted for wounds on the feet. In addition, pain was a commonly reported sequela.

## Discussion

In this systematic review, we sought to examine cosmetic outcome of wounds healed by SIH. We found that majority of wounds on the central median/temporal forehead, glabella, brow and suprabrow, medial canthus, lower eyelid, nasal ala, cheeks, upper lip and lower lip, postauricular area, and feet produced good to excellent cosmetic results. In contrast, wounds on the scalp, nasal dorsum, nasal tip, nasal sidewall, and chin (involving the periosteum) produced less impressive cosmetic results. These findings are aligned with prior published reports highlighting the importance of anatomic location in determining the healing outcome by SI.^44^ In addition to anatomic location, other important parameters reported in the literature include wound size and depth. In our study, the impact of wound size and depth on cosmetic outcome varied based on anatomic location. For instance, the cosmetic outcome of wounds on the nasal ala and upper eyelid correlated with wound depth but with minimal correlation with wound size. In contrast, there was no correlation between wound size, depth, and cosmetic outcome of wounds on the temple and antihelix. Furthermore, demographic characteristics such as age did not appear to influence wound outcome.

Consistent with prior reports, we found that postoperative complications with SIH did not differ significantly from those seen in wounds healed by primary closure. However, there were higher rates of infection in wounds in which cartilage was exposed and wounds on the feet.

In the appropriate clinical setting, SIH can be a cost-effective method of healing resulting in an acceptable cosmetic outcome. SIH may also enable a straightforward assessment of tumor recurrence. However, some of the disadvantages of SIH that limits its use in clinical practice include prolonged postoperative care, wound care time, and the psychosocial impact of caring for an open wound for an extended period.^45^

There are notable limitations to majority of the studies included in this review. Primarily, there was significant heterogeneity in the instruments used to measure cosmetic outcome. In addition, the lack of adequate definition of many nominal scales such as ‘good’ or ‘excellent’ makes it challenging to perform interstudy comparisons. The quality of the included studies also varied significantly, with only a few published reports being designed as randomized controlled trials. It is also important to appreciate that the baseline cosmetic desirability of outcomes of wounds in some of these locations may vary even with wounds that are closed primarily; thus, while scalp wounds may heal secondarily with a less than ideal scar, other repair options for larger wounds in this area—such as full thickness skin grafts—may also not be cosmetically ideal. Our approach was unable to capture this important distinction which would be best addressed in future prospective comparative studies. Finally, an additional important source of bias is the lack of randomization to SIH. Therefore, the observed success in treating a range of wounds with SIH may stem more from appropriate patient selection rather than a generalizable acceptability of treating a wide range of wounds with SIH. In addition, findings derived from the included surgical wounds cannot be extrapolated to other types of surgical excisions. Additional limitations inherent to the included studies were high attrition rates and varied follow-up times.

In summary, SIH appears to produce an acceptable cosmetic outcome for selected defects and may be of clinical benefit for a selected group of patients. In addition to its apparent cost-effectiveness (which must be weighed against the increased direct and indirect cost of caring for an open wound for an extended period) and low upfront time requirement for the clinician, the ability for SIH to produce an acceptable outcome makes it a useful adjunct in clinical practice, particularly in low-resource regions. Given the significant impact of anatomic location on SIH, however, and the wide range of reported levels of cosmetic acceptability in a range of anatomic locations, as well as the requisite time investment during the potentially lengthy healing process, appropriate patient selection is crucial.

## Conflicts of interest

None disclosed.
